# Dinosaur science

**DOI:** 10.1098/rsbl.2025.0297

**Published:** 2025-07-02

**Authors:** Paul M. Barrett, Susannah C. R. Maidment

**Affiliations:** ^1^ Natural History Museum, London, UK

**Keywords:** dinosaurs, evolution, palaeobiology

## 1. Introduction

In 1824, Reverend William Buckland, an eccentric cleric and groundbreaking geologist, presented a paper to the assembled ranks of the Geological Society of London. In it, he announced the discovery of a ‘great fossil lizard’ from the Jurassic-aged slate of Stonesfield, Oxfordshire, UK [1]. Although Buckland did not know it at the time, this animal—which he dubbed *Megalosaurus—*would later be recognized as the first named representative of what is now the most famous group of extinct animals. Shortly thereafter, two other giant land reptiles from southern England, *Iguanodon* and *Hylaeosaurus*, were named by Sussex doctor Gideon Mantell [2,3]. Building on these discoveries, the brilliant anatomist Sir Richard Owen realized that these three animals shared unusual features of their limbs and limb girdles that were not seen in other reptiles. Based on these features, Owen united this triumvirate in a new group—Dinosauria [4]. Since then, over 1300 valid species of dinosaur have been described, with their remains known from every continent [5]. Studying these animals has revolutionized our knowledge of the Mesozoic world in which they lived and shed light on the biological challenges of life at large body size. In addition, we now know that birds are an integral part of the dinosaur radiation, and studies of dinosaur palaeobiology are helping to elucidate the origins of many characteristic avian features, such as feathers [6].

To mark the 200th anniversary of the naming of *Megalosaurus,* we were invited to convene this Special Issue, which consists of 10 papers. Our aim was to assemble a series of contributions dealing with specific areas of dinosaur research, reviewing the state of the art and offering stimulus for future work. In particular, we encouraged the authors to offer their personal opinions on the *status quo* and to highlight current controversies or knowledge gaps in the field.

## 2. Quality of the record

Maidment & Butler [5] take a detailed look at the raw data underlying our work: the dinosaur fossil record. Using bibliographic data they assess how this resource has grown, discuss its underlying structure and look at broad-scale trends in dinosaur discovery. They note that discoveries of new dinosaur taxa continue apace, but there are many regions of the world (e.g. Africa and India) and time periods (e.g. the early Late Triassic and Middle Jurassic) that are undersampled. These gaps have significant potential to provide new material that would impact current scenarios of dinosaur diversification. They advocate the need for more foundational field and taxonomic work, which is often difficult to fund.

Taking another tack, Mannion [7] assesses the geological and anthropic sampling biases that affect the quality of the record and how these might influence reconstructions of macroevolutionary patterns (such as the trajectory of species richness through time, the magnitude of adaptive radiations and responses to extinction events). He shows that literal readings of the record are simplistic and should not be trusted, but introduces methodological approaches to mitigate some of these issues.

Dinosaur occurrence data also underpin the contribution of Upchurch & Chiarenza [8] on dinosaur palaeobiogeography. These authors use phylogenetic branching patterns, divergence dates and palaeogeography to assess the influence of geodispersal and vicariance on dinosaur evolutionary history. They show that vicariance played a significant role in dinosaur distribution, following the rifting sequence of the Pangaean supercontinent.

## 3. Palaeobiological insights

Dinosaur biology has intrigued scientists since the nineteenth century, but until comparatively recently attempts to reconstruct their life habits and physiology were often based on weak foundations. One of the major advances in recent decades has been the introduction of more rigorous, quantitative approaches, drawing on methods developed in other fields, including biological imaging and structural engineering, as well as a better understanding of living relatives and other analogues.

Chapelle *et al.* [9] summarize current knowledge of dinosaur growth and reproduction, an area that has benefitted greatly from recent discoveries of eggs, nests and juveniles, and from the widespread adoption of osteohistological techniques. Documenting the distributions and abundances of different tissue types and growth features in bone thin sections now allows accurate reconstruction of growth rates and ontogenetic stage, thereby offering new insights into dinosaur parenting, metabolism, taxonomy and community structure.

Whether dinosaurs were ectothermic, like their reptilian ancestors, or endothermic, like their living descendants, the birds, has long been debated and has fundamentally affected how palaeontologists have interpreted dinosaur lifestyles. Baumgart *et al.* [10] review the current evidence for dinosaur thermoregulation, metabolism, respiration and the cardiovascular and digestive systems. An important recommendation of this wide-ranging review is that a clear understanding of both intra- and interspecific variation is needed to accurately reconstruct the physiology of extinct animals, and that approaches based on the extant phylogenetic bracket of dinosaurs are often limited by comparisons with just a few individuals from exemplar species.

Dinosaur footprints are exceptionally abundant but garner less attention than skeletons in palaeobiological studies. However, Falkingham [11] shows that these traces can be used to provide vital data on dinosaur locomotion and reviews how new technologies can be used to extract exceptional detail from these fossils, which present direct windows on dinosaur behaviour. He also highlights new computational modelling methods that integrate musculoskeletal reconstructions and kinematics to gain new insights into how dinosaurs moved.

Drawing on a variety of palaeobiological and occurrence data, as well as cutting-edge evolutionary and ecological modelling methods, Chiarenza [12] discusses some of the macroecological patterns that shaped the clade’s evolution. These include trends in body size, the influence of palaeolatitudinal diversity gradients and potential floral coevolution, as well as the possible impacts of ‘general’ macroecological rules (Allen’s, Bergmann’s and island rules).

## 4. Dinosaurs and birds

The realization that birds are extant members of the dinosaur radiation revolutionized dinosaur research. It gave dinosaurs new relevance to biologists and provided a set of model organisms through which to interpret non-avian dinosaur behaviour and morphology.

Xu & Barrett [6] provide an overview of feather evolution, drawing on direct evidence from exceptionally preserved fossils and an increasing body of data drawn from evolutionary developmental biology. Although the dinosaurian origin of feathers is well established, many key issues still require resolution, including more agreement over what constitutes ‘a feather’ (rather than other similar epidermal structures) and how often and when these features appeared in dinosaur evolution.

Focusing on another major biological transformation, Balanoff [13] examines the evolution of the nervous system through the theropod–bird transition. This serves as a case study for how to interpret neuroanatomy from fossils, and highlights the major impacts of non-destructive, advanced imaging techniques as well as cutting-edge work on the neurobiology of living relatives.

Closing the volume, Field *et al.* [14] concentrate on the Mesozoic radiation of birds. They showcase a range of advances made possible by new specimens and the application of new methods, highlighting new solutions to long-standing problems relating to the evolution of the skull, shoulder girdle and postcranial pneumaticity. This sets the scene for the spectacular radiation of modern birds, with over 11 000 living species, and helps to remind us that dinosaurs are not extinct.

## Data Availability

This article has no additional data.
